# Acid Sphingomyelinase Controls Early Phases of Skeletal Muscle Regeneration by Shaping the Macrophage Phenotype

**DOI:** 10.3390/cells10113028

**Published:** 2021-11-05

**Authors:** Paulina Roux-Biejat, Marco Coazzoli, Pasquale Marrazzo, Silvia Zecchini, Ilaria Di Renzo, Cecilia Prata, Alessandra Napoli, Claudia Moscheni, Matteo Giovarelli, Maria Cristina Barbalace, Elisabetta Catalani, Maria Teresa Bassi, Clara De Palma, Davide Cervia, Marco Malaguti, Silvana Hrelia, Emilio Clementi, Cristiana Perrotta

**Affiliations:** 1 Department of Biomedical and Clinical Sciences “Luigi Sacco” (DIBIC), Università degli Studi di Milano, 20157 Milano, Italy; paulina.roux@unimi.it (P.R.-B.); m.coazzoli@gmail.com (M.C.); silvia.zecchini@unimi.it (S.Z.); ilaria.direnzo1989@gmail.com (I.D.R.); alessandra.napoli@unimi.it (A.N.); claudia.moscheni@unimi.it (C.M.); matteo.giovarelli@unimi.it (M.G.); emilio.clementi@unimi.it (E.C.); 2 Department for Life Quality Studies, Alma Mater Studiorum-University of Bologna, 47921 Rimini, Italy; pasquale.marrazzo2@unibo.it (P.M.); maria.barbalace2@unibo.it (M.C.B.); marco.malaguti@unibo.it (M.M.); silvana.hrelia@unibo.it (S.H.); 3 Department of Pharmacy and Biotechnology, Alma Mater Studiorum-University of Bologna, 40126 Bologna, Italy; cecilia.prata@unibo.it; 4 Department for Innovation in Biological, Agro-Food and Forest Systems (DIBAF), Università degli Studi della Tuscia, 01100 Viterbo, Italy; ecatalani@unitus.it (E.C.); d.cervia@unitus.it (D.C.); 5 Scientific Institute IRCCS “Eugenio Medea”, 23842 Bosisio Parini, Italy; mariateresa.bassi@lanostrafamiglia.it; 6 Department of Medical Biotechnology and Translational Medicine (BIOMETRA), Università degli Studi di Milano, 20129 Milano, Italy; clara.depalma@unimi.it

**Keywords:** acid sphingomyelinase, muscle regeneration, macrophage phenotype, inflammation

## Abstract

Skeletal muscle regeneration is a complex process involving crosstalk between immune cells and myogenic precursor cells, i.e., satellite cells. In this scenario, macrophage recruitment in damaged muscles is a mandatory step for tissue repair since pro-inflammatory M1 macrophages promote the activation of satellite cells, stimulating their proliferation and then, after switching into anti-inflammatory M2 macrophages, they prompt satellite cells’ differentiation into myotubes and resolve inflammation. Here, we show that acid sphingomyelinase (ASMase), a key enzyme in sphingolipid metabolism, is activated after skeletal muscle injury induced in vivo by the injection of cardiotoxin. ASMase ablation shortens the early phases of skeletal muscle regeneration without affecting satellite cell behavior. Of interest, ASMase regulates the balance between M1 and M2 macrophages in the injured muscles so that the absence of the enzyme reduces inflammation. The analysis of macrophage populations indicates that these events depend on the altered polarization of M1 macrophages towards an M2 phenotype. Our results unravel a novel role of ASMase in regulating immune response during muscle regeneration/repair and suggest ASMase as a supplemental therapeutic target in conditions of redundant inflammation that impairs muscle recovery.

## 1. Introduction

Skeletal muscle regeneration is a well-orchestrated process of response to damage followed by the degeneration of myofibers and acute inflammation [1,2,3,4]. The key players in the reconstruction of an injured muscle are satellite cells, muscle stem cells that are quiescent in a healthy muscle and start to proliferate and differentiate after damage [2,4]. The inflammatory response is initially required for a positive outcome of muscle repair since specialized immune cells are necessary for removing myofiber debris and producing cytokines that trigger satellite cell function [5,6,7]. However, a persistent inflammation limits the regenerative capacity of satellite cells and induces muscle wasting thus impairing muscle recovery [8,9].

Macrophage cells are the major players regulating inflammation and regeneration in muscles [10,11,12] since they can assume different phenotypes according to the microenvironment of inflamed tissue [13]. The activation state of macrophages shapes myogenesis by the interaction with myogenic precursor cells. In the initial stage after damage, the release of inflammatory cytokines such as interferon γ (IFN-γ) and tumor necrosis factor α (TNF-α) drives the activation of macrophages to a proinflammatory phenotype (M1) that perpetuates inflammation and promotes myoblast proliferation [14,15]. Later, M1 macrophages are replaced by a population of anti-inflammatory and tissue repairing macrophages (M2) that enhance differentiation of satellite cells and their fusion into myotubes [16,17]. Thus, the transition of the immune microenvironment from the former dominated by M1 macrophages to the latter characterized by M2 macrophages appears to be functionally coupled to the myogenesis stages.

Understanding the cellular/molecular players determining macrophages phenotype in injured muscle and the mechanisms underlying M1 to M2 switch may indicate therapeutic interventions to ameliorate inflammation and boost functional muscle recovery after damage by affecting the balance between M1 and M2 macrophages.

Several inflammation-related pathologies are accompanied by an abnormal expression and activation of acid sphingomyelinase (ASMase) [18,19,20,21,22,23,24,25], one of the enzymes responsible for the hydrolysis of sphingomyelin and the ensuing generation of ceramide. Ceramide is crucial for normal tissue homeostasis since it acts as second messenger in cellular signaling, but it is detrimental when produced at altered concentrations [26,27,28,29,30,31,32]. ASMase is activated by stress stimuli, as for instance lipopolysaccharide (LPS), TNF-α, and interleukin (IL) 1β [33,34,35,36,37]; in turn, expression/activity of ASMase regulates the levels of key mediators such as IL1β and IL6 in different pathological conditions [38,39,40,41].

Using the ASMase-knock out (KO) mice, in this study, we unravel a novel role for ASMase in muscle regeneration, associated with its involvement in inflammatory response. The regeneration capacity of ASMase-KO skeletal muscle was assessed after cardiotoxin (CTX) injection, in which muscle degeneration occurs early after damage and inflammatory cells are quickly recruited at the injured site [42,43]. Our data demonstrate that ASMase while not affecting satellite cells proliferation and differentiation *per se* controls muscle regeneration at early stages by shaping macrophages phenotypes and modulating inflammation.

## 2. Materials and Methods

### 2.1. Animals and Muscle Injury Model

ASMase-KO mice (C57BL/6N strain) [44] were generated crossing heterozygous mice and were always compared to wild-type (WT) littermates used as control. Genotypes were checked by PCR [44]. All animals were housed in pathogen-free conditions. All procedures were carried out in strict accordance with the Italian law on animal care (D.L. 26/2014, implementation of the 2010/63/UE) and approved by University of Milan Animal Welfare Body and by the Italian Minister of Health (n° 924/2018-PR). All efforts were made to reduce both animal suffering and the number of animals used.

The analysis of skeletal muscles (tibialis anterior, gastrocnemius, quadricep and diaphragm) were performed in male and female of 1, 2 and 3 months of age.

Acute muscle damage was induced by injection of CTX from *Naja pallida* (50 μL, 10 μM, Latoxan, Portes-lès-Valence, France), in the tibialis anterior (TA) of 2.5 months old, anesthetized mice [42]. Mice were sacrificed at 1, 3, 5, 7, 14, and 21 days after injury. Injured muscles were collected and snap-frozen in liquid nitrogen for RNA analyses. For histology, muscles were collected and directly frozen in liquid nitrogen-cool isopentane and stored at −80 °C until processed.

### 2.2. Primary Cell Isolation and Culture

Satellite cells from 1 month old WT and ASMase-KO mice were obtained from muscles of hindlimbs and forelimbs by using the tissue dissociation protocol of gentleMACS™ Octo Dissociator with Heaters (Miltenyi Biotec, Bergisch Gladbach, Germany) followed by magnetic depletion of lineage PECAM1-, PTPRC-, ITGAM-, and LY6A/Sca-1-positive cells using the Satellite Cell Isolation Kit (Miltenyi Biotec), according to the manufacturer’s protocols [45].

Satellite cells were cultured in DMEM (EuroClone, Milan, Italy) supplemented with 20% fetal bovine serum (FBS) (EuroClone,), 3% chick embryo extract (United States Biological, Salem, MA, USA), 10 ng/mL basic fibroblast growth factor (FGFb) (PeproTech, Cranbury, NJ, USA) and 1% penicillin-streptomycin (EuroClone) on Matrigel-coated (Corning, New York, NY, USA) plates at 37 °C with 5% CO_2_ for 4 days. To assess proliferation, satellite cells were plated at a confluence of 1.5 × 10^4^ cell/cm^2^ in growth medium and cultured for 24 h. For the differentiation experiment, cells were plated at a confluence 5 × 10^4^ cell/cm^2^ in medium containing 2% Horse Serum (EuroClone) instead of FBS and cultured for 48 h [1].

Bone marrow precursor cells (MONO) were harvested by flushing femurs and tibiae of 2–3 month old WT and ASMase-KO mice and cultured for 5 days in α-MEM (37 °C, 5% CO_2_ in a humidified atmosphere) containing 10% FBS and of 100 ng/mL macrophage-specific colony-stimulating factor (M-CSF) (Miltenyi Biotec) to generate macrophages. Cells were cultured for 2 additional days in the presence of 10 ng/mL of M-CSF and 50 ng/mL IFN-γ (Miltenyi Biotec) to generate M1 cells and for 4 additional days with 10 ng/mL M-CSF and 10 ng/mL IL4 (Miltenyi Biotec) to generate M2a cells [24,46].

To assess the effect of pharmacological inhibition of ASMase on macrophage differentiation/polarization, amitriptyline hydrochloride (Sigma-Aldrich, Saint Louis, MO, USA) [27] at the final concentration of 5 μM were added to WT cells on the days 0, 3 and 5 of culture.

### 2.3. Histology and Immunofluorescence

Hematoxylin-eosin (H&E, Bio Optica, Milan, Italy) staining and immunofluorescence were performed on 7 µm-thick serial muscle sections obtained with a cryostat [47].

For immunofluorescence, sections were fixed for 10 min with 4% paraformaldehyde (PFA) in PBS and then blocked with 10% normal goat serum (Sigma-Aldrich) and 0.1% Triton X-100 (Sigma-Aldrich) in PBS for 1 h. All primary antibodies were diluted in blocking solution and incubated overnight at 4 °C. After incubation with the appropriate fluorescent-labeled secondary antibodies diluted in blocking solution for 1 h (Alexa Fluor conjugated antibodies; ThermoFisher, Walthan, MA, USA), nuclei were counterstained with DAPI (PureBlu, Bio-Rad, Hercules, CA, USA) and slides were finally mounted with the Fluoroshield Histology Mounting Medium (Sigma-Aldrich) [48]. To measure the cross-sectional area (CSA) of myofibres, muscle sections were stained with an anti-laminin antibody (Sigma-Aldrich). The ImageJ software was used to determine the CSA of 1000 to 3000 individual fibers from at least three different fields for each muscle section. Four to nine sections from each muscle were analyzed. The other antibodies used were: embryonal myosin heavy chain (MyHC-Emb; Santa Cruz Biotechnology, Dallas, TX, USA), CD45 (Miltenyi Biotec), CD80 and CD206 (BioLegend, San Diego, CA, USA), MyoD (Agilent Dako, Santa Clara, CA, USA) and Ki67 (Abcam, Cambridge, UK) [49,50].

For cultured satellite cells staining, cells were fixed with 4% PFA for 10 min at room temperature and permeabilized with 0.1% Triton X-100 in PBS for 5 min at room temperature. Cells were then blocked with 10% normal goat serum in PBS and labeled with the primary antibodies Ki67, in proliferating satellite cells, and myosin heavy chain (MyHC)—MF20; Developmental Studies Hybridoma Bank), in differentiated myotubes in blocking solution at 4 °C overnight [45,51]. Cells were then incubated with Alexa Fluor-conjugated antibodies in blocking solution for 1 h at room temperature. Image analysis was performed by using ImageJ software. Fusion index, diameter of myotubes, number of nuclei/myotubes and myotubes ≥ 5 nuclei were calculated from five to ten randomly chosen microscopic fields. Fusion index was calculated as the percentage of number of nuclei within myotubes over the total number of nuclei.

Images were acquired using a DMI4000 B fluorescence microscope Leica automated inverted microscope equipped with a DCF310 digital camera (Leica Microsystems, Wetzlar, Germany) or the ZOE™ Fluorescent Cell imager (Bio-Rad) and the Leica TCS SP8 System equipped with Leica DMi8 inverted microscope, for confocal imaging.

### 2.4. Whole Body Tension

The whole body tension (WBT) assay was used to determine the ability of mice to exert tension in a forward pulling maneuver that is elicited by stroking the tail of the mice [1,52]. The tails were connected to an MP150 System transducer (BIOPAC Systems, Goleta, CA, USA) with a 4.0 silk thread (one end of the thread being tied to the tail and the other end to the transducer). Mice were placed into a small tube constructed of a metal screen with a grid spacing of 2 mm and exerted a small resting tension on the transducer. Forward pulling movements were elicited by a stroke of the tail with serrated forceps and the corresponding tensions were recorded using a AcqKnowledge software recording system (BIOPAC Systems). Between 20 and 30 pulling tensions were recorded during each session. The WBT was determined by dividing the average of the top five or top ten forward pulling tensions by the body weight and represent the maximum phasic tension that can be developed over several attempts.

### 2.5. Flow Cytometry

Tibialis anterior muscles from WT and ASMase-KO mice were harvested, minced and digested in a freshly prepared solution containing dispase and type II collagenase for 40 min at 37 °C. Disaggregation was stopped with 10% FBS and cells filtered through a 70 μm cell strainer (Miltenyi Biotec). The collected cells were washed with PBS supplemented with 2% FBS and incubated with primary conjugated antibodies for 30 min at 4 °C and analyzed by using Gallios Flow Cytometer (Beckman-Coulter, Brea, CA, USA) and the software FCS Express 4 (De Novo System, Portland, OR, USA). Satellite cells were identified as an enriched population of α7-Integrin-PE (AbLab, Vancouver, BC, Canada) and CD34-Alexa Fluor 647 (BD Pharmingen™, San Diego, CA, USA) double-positive cells and CD45-PE-Cy7, CD31-PE-Cy7, CD80-FITC, CD86-FITC, CD14-FITC (eBioscience, San Diego, CA, USA) and Sca1-FITC (BD Pharmingen™) negative cells [53]. M1 macrophages were identified as CD45-PE-Cy7, F4/80-PE, CD80-FITC positive cells; M2 macrophages as CD45-PE-Cy7, F4/80-PE, CD206-APC positive cells [23,24].

### 2.6. ASMase Activity Assay

The activity of ASMase was assessed on both muscle and macrophage homogenates obtained by using Ultra-Turrax^®^ homogenator (T 10, IKA, Wilgminton, NC, USA) followed by 3 freeze-thaw cycles, and sonication for 10 s at 55% power (Sonopuls Ultrasonic homogenizer HD 2070, Bandelin, Berlin, Germany) in water. Homogenates (30 µg per sample) were assayed for ASMase activity using the ASMase assay kit (Echelon Biosciences, Salt Lake City, UT, USA) following the manufacturer’s protocol. Fluorescence analysis was performed using a GloMax-Multi detection system plate reader (Promega, Madison, WI, USA; excitation: 365 nm, emission 410–460 nm).

### 2.7. RNA Extraction and RT-qPCR

The analysis of mRNA expression was performed as previously described [24,54,55,56]. Total RNA from muscles, satellite cells and macrophages was isolated by phase separation in PureZOL reagent (Bio-Rad) according to the manufacturer’s instructions. After solubilization in RNase-free water, total RNA was quantified by Nanodrop 2000 spectrophotometer (ThermoFisher). Total RNA (800–1000 ug) was retro-transcribed using iScript gDNA Clear cDNA Synthesis Kit (Bio-Rad). RT-qPCR was performed using SsoAdvanced™ Universal SYBR Green Supermix (Bio-Rad) and the CFX96 Touch Real-Time PCR Detection System (Bio-Rad). RPL32, RPL38 and 36B4 have been used as housekeeping genes for normalization by using the 2^−ΔΔCT^. The primers pairs designed for RT-qPCR are detailed in Table 1.

### 2.8. Statistical Analysis

Statistical significance of raw data between the groups was evaluated using unpaired Student’s *t* test (single comparisons) or one-way ANOVA followed by Bonferroni or Tukey post-tests (multiple comparisons) or two-way ANOVA with correction for multiple comparisons using the Sidak test (grouped analysis). When data are not normally distributed, the Mann-Whitney test was used. The analysis was carried out by using GraphPad Prism software package (GraphPad Software, San Diego, CA, USA). The results are expressed as means ± SEM of the indicated *n* values. *p* values ≤ 0.05 were considered statistically significant.

## 3. Results

### 3.1. ASMase Ablation Does Not Affect the Phenotype/Function of Skeletal Muscle and the Content of Satellite Cells

**Figure 1:**
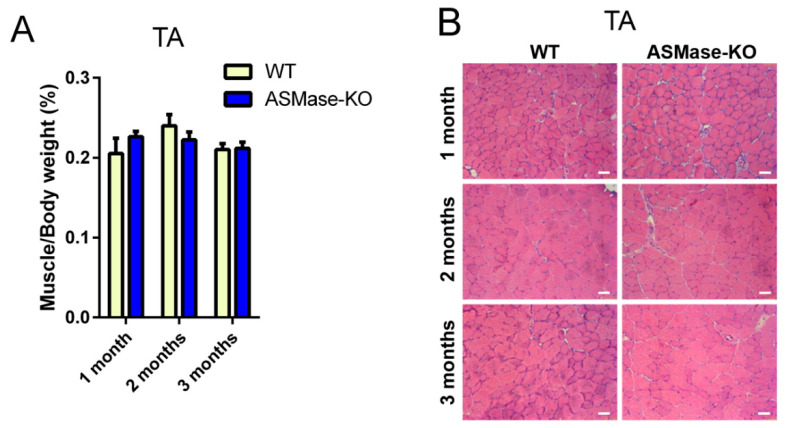

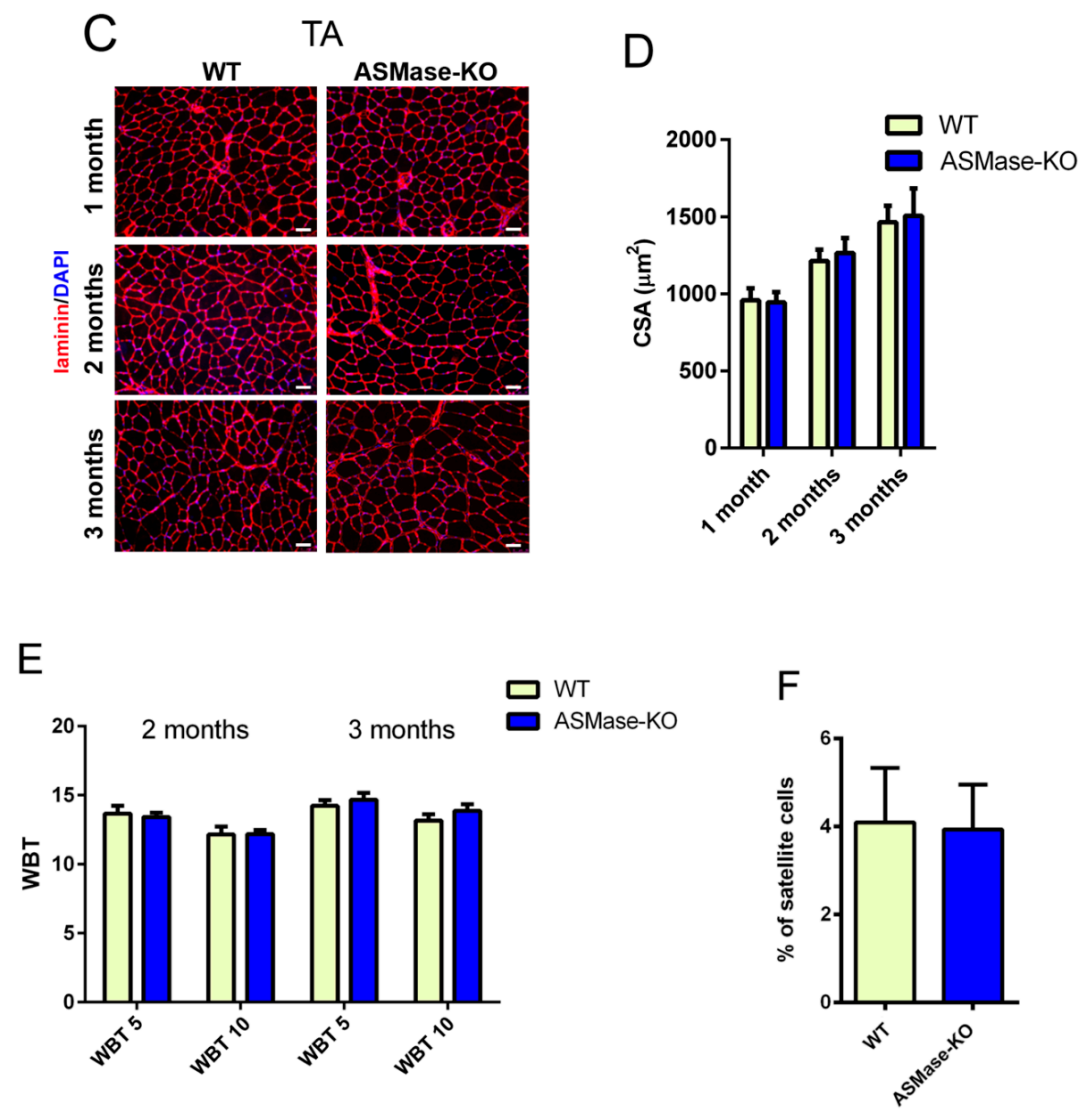
Characterization of skeletal muscle phenotype and function in ASMase-KO mice. (**A**) Muscle weight, normalized to body weight, of TA muscles in 1 month, 2 months and 3 months old WT and ASMase-KO mice (*n* ≥ 4 mice). (**B**,**C**) Representative images of H&E staining (**B**), and Laminin (red) and DAPI (Blue), for the nuclei, (**C**) immunostaining of transverse sections of TA of 1 month, 2 months and 3 months old WT and ASMase-KO mice. Scale Bar, 50 μm. (**D**) Quantification of mean CSA of TA of 1 month, 2 months and 3 months old WT and ASMase-KO mice (*n* ≥ 4 mice) measured using ImageJ based on laminin staining. (**E**) WBT measurements determined by dividing the average of the top ten or top five forward pulling tensions, respectively, by the body weight in 1 month, 2 months and 3 months old WT and ASMase-KO mice (*n* = 10). (**F**). Satellite cell quantification by flow cytometry in TA muscles of 3 months old WT and ASMase-KO mice (*n* = 3 mice).

We first analyzed skeletal muscles of WT and ASMase-KO mice, namely the TA, gastrocnemius (GAS), quadriceps (QUAD), and diaphragm (DIA), at different ages (1, 2 and 3 months) to evaluate muscle growth. The weight and gross morphology of muscles in ASMase-KO mice were indistinguishable from those of their WT littermates at all ages analyzed (Figure 1A and Supplementary Figure S1A,B). Histological analysis by H&E did not show any overt abnormalities in ASMase-KO mice, including no signs of degeneration (Figure 1B and Supplementary Figure S1C). Moreover, CSA measuring the fiber size did not reveal any significant difference between muscles of the two genotypes (Figure 1C,D and Supplementary Figure S1D,E).

In agreement with a normal muscle phenotype, in ASMase KO mice the WBT normalized for body weight did not differ at any age from that observed in the WT controls (Figure 1E), indicating a preserved in vivo muscle force in the absence of ASMase.

We also assessed the number of satellite cells in muscles of 3 months old WT and ASMase-KO mice by flow cytometry finding a similar percentage of satellite cells in both genotypes (Figure 1F and Supplementary Figure S2). All these data indicate that the loss of ASMase neither affects the normal skeletal muscle morphology and function nor satellite cell number in healthy muscles.

### 3.2. ASMase Ablation Does Not Affect the Proliferation and Differentiation of Satellite Cells

**Figure 2:**
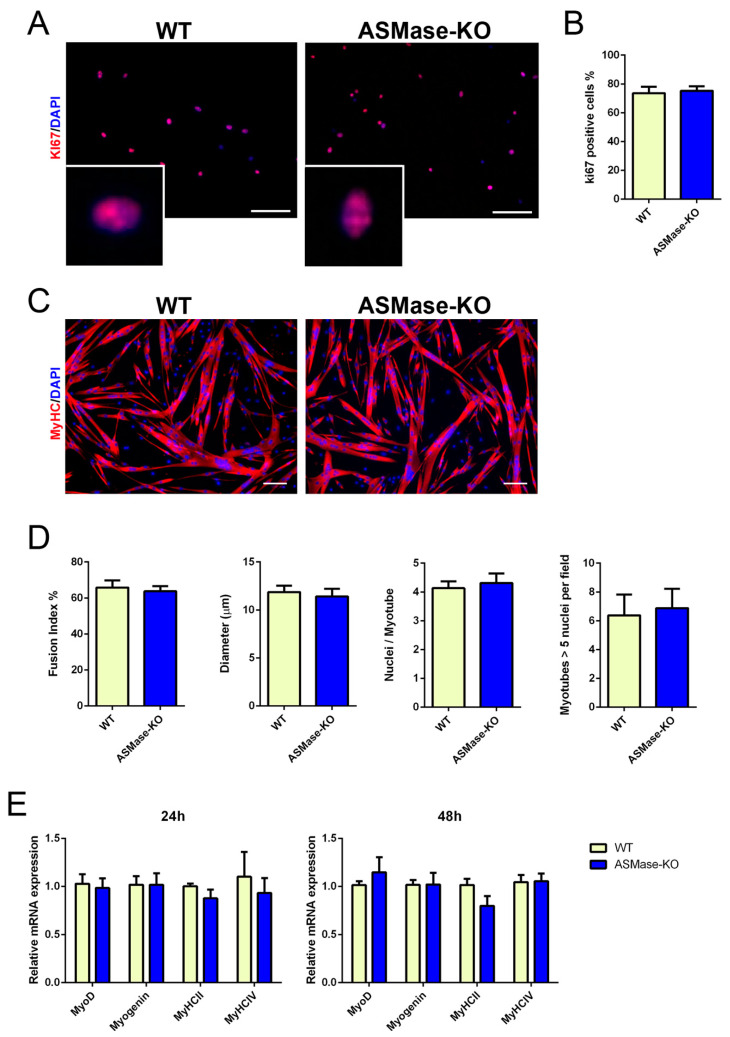
ASMase in satellite cells proliferation and differentiation in vitro. (**A**) Representative Ki67 immunostaining (red) and DAPI nuclear counterstaining (blue) of satellite cells isolated from WT and ASMase-KO mice and cultured for 24 h. Scale bar, 100 µm. (**B**) Percentage of Ki67 positive satellite cells on total DAPI staining. Values are expressed as mean ± SEM (*n* = 5 mice). (**C**) Representative MyHC immunostaining (red) and DAPI nuclear counterstaining (blue) of satellite cells isolated from WT and ASMase-KO mice and cultured for 48 h. Scale bar, 100 µm. (**D**) Fusion index, mean myotubes diameter, mean number of myonuclei/myotube and percentage of myotubes with 5 or more nuclei of satellite cells from WT and ASMase-KO mice (*n* = 9 mice). (**E**) RT-qPCR analysis of myogenic markers Myod, Myog, and MyHCII and MyHCIV (*n* ≥ 4 mice).

As satellite cells are able to proliferate and differentiate in response to injury giving rise to regenerated muscle, we wondered whether the absence of ASMase would affect these capabilities. Thus, we established primary muscle progenitor cell culture from WT and ASMase-KO mice and compared the in vitro proliferation and differentiation ability by immunostaining with the cell cycle marker Ki67 and the marker of myotubes myosin-heavy-chain (MyHC), respectively. Proliferation of myoblasts, measured after 24 h of culture, was similar between the two genotypes (Figure 2A,B). No differences were likewise observed in single myoblast fusion to form nascent myotubes and its following growth after 48 h of culture to lead larger fully differentiated myotubes assessed by parameters such as fusion index, myotube diameters, number of myonuclei per myotube and myotubes with five or more nuclei (Figure 2C,D).

We also examined the expression levels of the transcription factors MyoD and Myogenin, fundamental for myogenesis, and MyHCII and IV, responsible for muscle contraction, revealing no alterations in ASMase-KO derived cells compared to those obtained from WT mice (Figure 2E). Hence, our data indicate that the lack of ASMase is not required for proper satellite cells functions outside the regenerating muscle niche.

### 3.3. ASMase Is Activated in CTX-Induced Acute Damage and Its Loss Speeds Early Skeletal Muscle Regeneration

Since ASMase exerts an important role in promoting and modulating immune response [38,39,40,41], we assessed its role in muscle inflammation/regeneration by injecting TA with CTX in WT and ASMase-KO mice.

**Figure 3:**
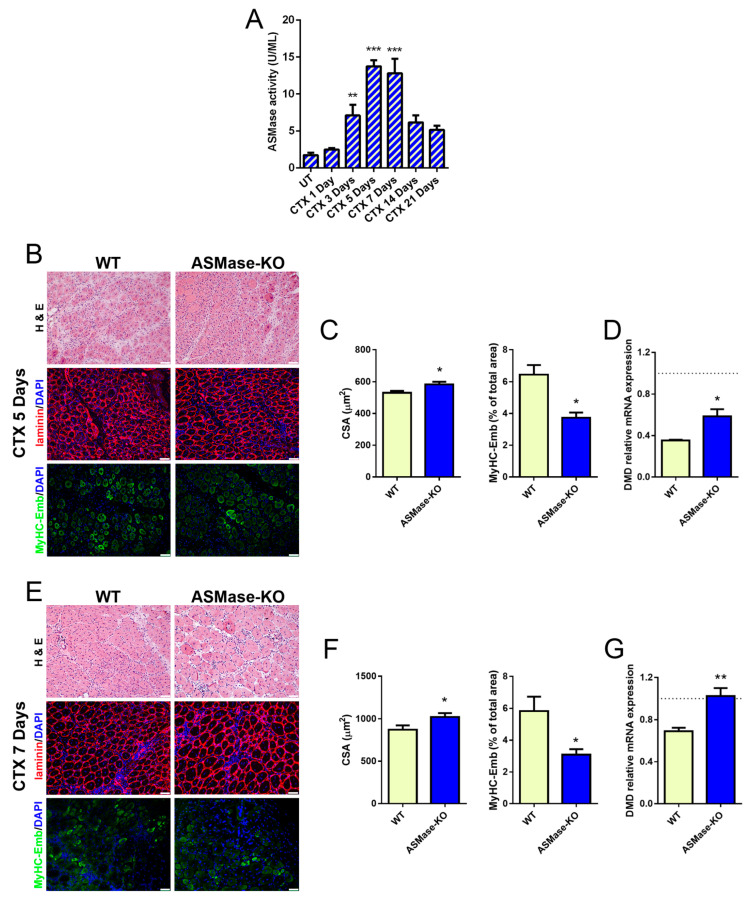
ASMase in CTX-induced injury. (**A**) Acid sphingomyelinase activity measured at different time points after CTX injection in tibialis anterior of WT mice (*n* ≥ 4 mice). Values are expressed as mean ± SEM. ** *p* < 0.01, *** *p* < 0.001 vs. the untreated control (UT). (**B**–**D**) Analysis of TA muscles at 5 days after CTX injection in WT and ASMase-KO mice. (**B**) Representative images of H&E staining (top panels), laminin (middle panels) and MyHC-Emb (bottom panels) immunostaining of transverse sections of TA muscles. Nuclei were counterstained with DAPI (blue). Scale bar, 50 μm. (**C**) Mean CSA quantification (left graph) of regenerating fibers (centrally nucleated) measured on laminin staining (*n* = 3 mice) and MyHC-Emb quantification (right graph) (*n* = 4 mice) of TA muscles. Values are expressed as mean ± SEM. * *p* < 0.05 vs. the WT. (**D**) RT-qPCR analysis of DMD. Values are expressed as mean ± SEM (*n* = 3 mice) normalized vs. the untreated controls (dashed line). * *p* < 0.05, vs. the WT. (**E**–**G**) Analysis of TA muscles at 7 days after CTX injection in WT and ASMase-KO mice. (**E**) H&E staining (top panels), laminin (middle panels) and MyHC-Emb (bottom panels) immunostaining of transverse sections of TA. Nuclei were counterstained with DAPI (blue). Scale bar, 50 μm. (**F**) CSA quantification (left graph) of regenerating fibers (centrally nucleated) measured on laminin staining (*n* = 3 mice) and MyHC-Emb quantification (right graph) (*n* = 5 mice) of TA muscles. Values are expressed as mean ± SEM. * *p* < 0.05 vs. the WT. (**G**) RT-qPCR analysis of DMD. Values are expressed as mean ± SEM (*n* = 3 mice) normalized vs. the untreated controls (dashed line). * *p* < 0.05, ** *p* < 0.01 vs. the WT.

First, we evaluated ASMase activity at various time points after CTX damage in TA of WT mice (Figure 3A). An increase of ASMase activity was observed starting from 3 days after the injection peaking at 5 and 7 days, suggesting that ASMase plays somewhat a role in the process of muscle regeneration in vivo.

To test this hypothesis, we evaluated the CSA of regenerating centrally nucleated fibers, and the expression of embryonal myosin (eMyHC) as a proxy for muscle regeneration upon CTX injury, in WT and ASMase-KO mice, at the time points corresponding to the highest ASMase activation, i.e., 5 and 7 days, in which regenerative myogenesis and tissue remodeling occur [2]. As shown in Figure 3B,C and in Figure 3E,F, ASMase-KO mice showed larger regenerating myofibers compared to WT and the expression of eMyHC at the site of injury was significantly decreased at both 5 and 7 days post-damage, indicating an enhanced growth of the new fibers as a result of faster myogenesis. In the same temporal window, the mRNA expression of dystrophin (DMD), which reflects accurately the extent of muscle regeneration [57], was higher in ASMase-KO when compared with WT muscles (Figure 3D,G). Similar results were obtained with myozenin1 (Myoz1) gene (Supplementary Figure S3A), further indicating increased myofiber maturity in the absence of ASMase. To note, in both type of mice no differences were found in the percentage of centrally nucleated fibers in the damaged area (Supplementary Figure S3B).

The increase of satellite cells in ASMase-KO mice at 3 and 5 days after damage in comparison to WT mice further supported these results (Supplementary Figure S3C,D). The effect observed was transient since fiber size was similar in both genotypes the at 14 days after injury (Supplementary Figure S3E). Taken together these data indicate that early muscle regeneration is accelerated in ASMase-KO mice even if ASMase does not directly affect satellite cells myogenic properties.

### 3.4. ASMase Modulates the Generation of Inflammatory Signals at the Injury Site

Inflammatory cells and their extrinsic factors play a significant role in creating the local environment at the site of muscle injury thus influencing satellite cells response to muscle damage; we investigated whether ASMase impacts on the immune response occurring in injured TA muscles.

**Figure 4:**
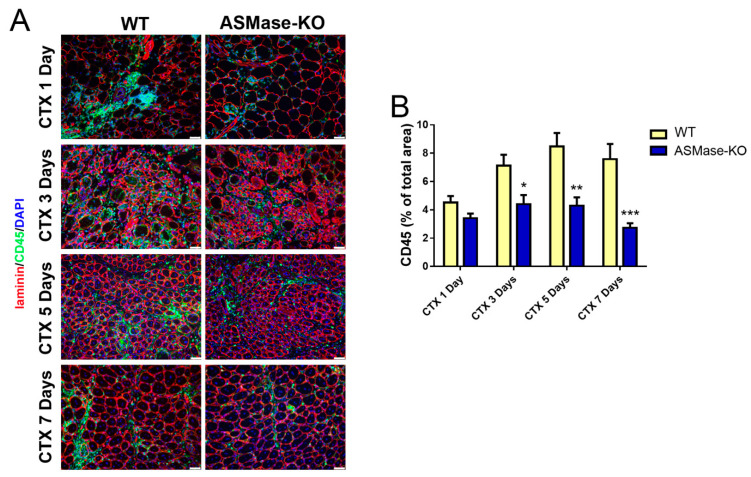

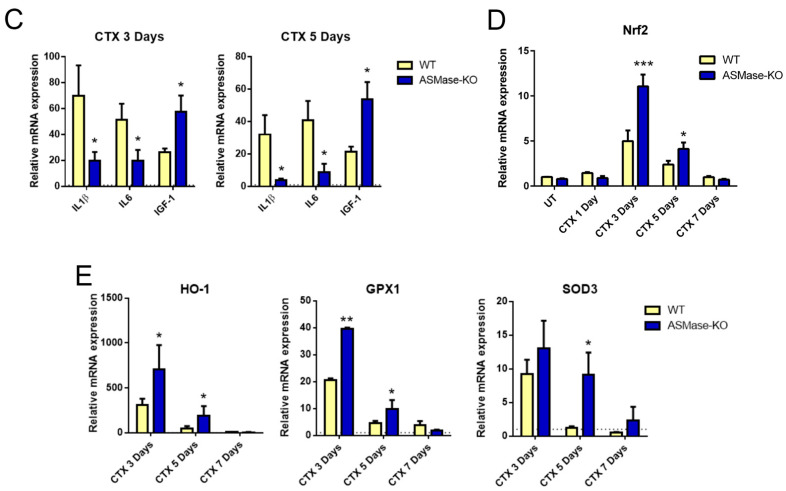
ASMase in inflammation in injured muscle. (**A**) Representative images of CD45 immunostaining and DAPI nuclear counterstaining (blue) of transverse sections from WT and ASMase-KO TA muscles at different time points after CTX injection. (**B**) CD45 positive (CD45+) quantification. Values are expressed as mean ± SEM (*n* = 3 mice). * *p* < 0.05, ** *p* < 0.01, *** *p* < 0.001 vs. the respective WT. (**C**) RT-qPCR analysis of inflammatory markers IL1β, IL6 and IGF-1 at 3 (left graph) and 5 (right graph) days after CTX injection. Values are expressed as mean ± SEM (*n* ≥ 4 mice) normalized vs. the untreated controls (dashed line). * *p* < 0.05 vs. the respective WT. (**D**) RT-qPCR analysis of Nrf2 at different time points after CTX injection. Values are expressed as mean ± SEM (*n* ≥ 3 mice) normalized vs the untreated control. * *p* < 0.05, *** *p* < 0.001 vs. the respective WT control. UT: untreated (**E**) RT-qPCR analysis of HO-1, GPX1 and SOD3 at different time points after CTX injection. Values are expressed as mean ± SEM (*n* ≥ 3 mice) normalized vs. the untreated controls (dashed line). * *p* < 0.05, ** *p* < 0.01 vs. the respective WT.

The reduced leukocyte infiltration, assessed by counting CD45+ cells at the site of injury (Figure 4A,B), as well as the lower expression of the proinflammatory cytokines IL1β and IL6 (Figure 4C) observed in ASMase-KO mice after CTX injection at different time points, demonstrated that the absence of ASMase modified the course of inflammation. That this event shapes a definite niche at the damaged site improving muscle regeneration was suggested by the concomitant increase of Insulin-like growth factor 1 (IGF-1) (Figure 4C), a potent enhancer of tissue regeneration [58,59].

The transcription factor nuclear factor erythroid 2-related factor 2 (Nrf2) and its signaling play a key role in protecting cells from oxidative stress and inflammation [60,61,62,63]. In this respect, the anti-inflammatory effect in the absence of ASMase was confirmed by the enhanced expression of Nrf2 (Figure 4D) and its downstream genes Heme oxygenase-1 (HO-1), Glutathione peroxidase 1 (GPX1) and Superoxide dismutase 3 (SOD3) (Figure 4E) in ASMase-KO mice following tissue damage. Of notice, no modification of Nrf2 was observed when comparing satellite cells derived from WT and ASMase-KO mice (Supplementary Figure S4A), indicating a specific effect on immune cells recruited at the injured muscle.

### 3.5. In the Absence of ASMase Macrophages Polarization Is Shifted towards an M2 Phenotype

**Figure 5:**
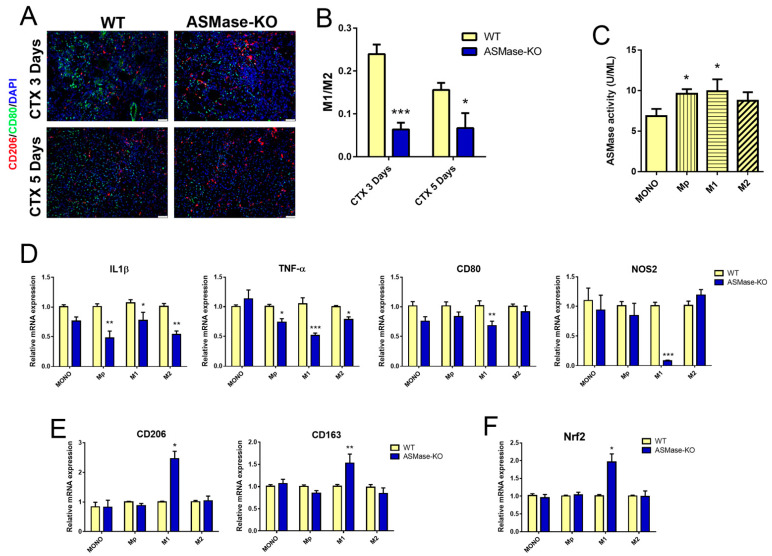
ASMase in macrophage differentiation and polarization. (**A**) Representative images of CD206 (red) and CD80 green immunostaining and DAPI nuclear counterstaining (blue) of transverse sections from WT and ASMase-KO TA muscles at 3 and 5 days after CTX injection. (**B**) M1/M2 ratio quantification by flow cytometry in TA muscles at 3 and 5 days after CTX injection in WT and ASMase-KO mice (*n* ≥ 3 mice for each group). Values are expressed as mean ± SEM normalized vs. the untreated controls (dashed line). * *p* < 0.05, *** *p* < 0.001 vs. the respective WT. (**C**) ASMase activity measured at different stages of macrophages differentiation and polarization, i.e., monocites (MONO), differentiated macrophages (Mp), M1-polarized macrophages (M1) and M2-polarized macrophages (M2) from WT mice. Values are expressed as mean ± SEM (*n* ≥ 3 mice). * *p* < 0.05 vs. MONO. (**D**–**F**) RT-qPCR analysis of the M1 markers IL1β, TNF-α, CD80 and NOS2 (**D**), the M2 markers CD206, and CD163 (**E**) and Nrf2 (**F**) in MONO, Mp, M1 and M2 from WT and ASMase-KO mice. Values are expressed as mean ± SEM (*n* ≥ 3 mice) normalized vs the untreated control. *p* < 0.05, ** *p* < 0.01, *** *p* < 0.001 vs. the respective WT.

Then, we analyzed the macrophage subsets in TA of WT and ASMase-KO mice after CTX injection by using CD80 and CD206 as cell surface markers for M1 and M2 macrophages, respectively, in immunofluorescence and flow cytometry analyses. A lower positivity to CD80 was observed in muscles from ASMase-KO, while no difference was observed in CD206 positive cells (Figure 5A and Supplementary Figure S4B). Accordingly, the M1/M2 ratio indicated that the proportion of M1 macrophages was significantly lower in the absence of ASMase (Figure 5B).

To further understand how the absence of ASMase alters the macrophage subsets balance in damaged muscles, we analyzed bone marrow-derived macrophages from WT and ASMase-KO in vitro.

First, ASMase activity was assessed in macrophages obtained from WT mice. As shown in Figure 5C, a significant increase in activity was observed in differentiated macrophages (Mp) and M1 polarized macrophages with respect to MONO, while no differences were detected in M2 polarized macrophages. The unchanged expression of Mp marker F4/80 in WT and ASMase-KO (Supplementary Figure S4C) revealed that the ASMase-KO derived cells differentiate normally, suggesting that ASMase does not affect macrophage differentiation.

Then, to evaluate a role for the enzyme in macrophage polarization, we analyzed different key genes expressed by the M1 and M2 subsets. Interestingly, we observed that ASMase-KO macrophages cultured in M1-polarizing conditions have a significantly lower expression of the M1 markers IL1β, TNF-α, CD80 and nitric oxide synthase 2 (NOS2) in comparison to the WT counterparts (Figure 5D). Similar results were obtained, at least in part, in M2 ASMase-KO macrophages. Notably, WT M1 macrophages treated with amitriptyline, a known functional inhibitor of ASMase (FIASMA) that blunts the enzyme activity, induced a significant reduction of IL1β, TNF-α and CD80 levels (Supplementary Figure S4D,E).

While no differences were revealed for CD206 and CD163 in ASMase-KO macrophages cultured in M2-polarizing conditions, we detected a significantly increased expression of these M2 markers in ASMase-KO M1 macrophages (Figure 5E). Taken together these data paralleled with the increase of Nrf2 expression in M1 macrophages lacking ASMase (Figure 5F), thus indicating a possible involvement of this transcription factor in the molecular machine triggered by ASMase.

## 4. Discussion

Skeletal muscle has an innate ability to repair after injury and heal spontaneously. However, severe muscle injuries can lead to the formation of fibrotic tissue that can impair muscle function. Thus, several strategies aimed at improving muscle recovery have been under investigation in the last decades [64].

In this study, we provide evidence for a functional role of ASMase in acute muscle damage. In mice bearing a functioning ASMase (WT), we observed that the enzyme is transiently activated upon CTX injection, during the phases of inflammation and regeneration [2], thus suggesting the connection of ASMase with these stages.

Myofiber repair, as well as growth during postnatal life, relies on the activation of satellite cells residing between the myofiber plasmalemma and basal lamina [4]. Sphingolipids play an essential structural role, especially in cell membranes, and can modulate multiple cell functions, such as proliferation, differentiation, mobility, and survival [65]. Among the sphingolipids derivatives, the ceramide/S1P rheostat has been shown to regulate the growth and differentiation of skeletal muscle cells [66,67,68]. In experiments carried out in vitro in the L6 muscle cell line ceramide, generated through the de novo synthesis, appears to negatively regulate myogenic differentiation [68]. Our data, obtained by analyzing satellite cells from ASMase-KO mice in vitro and in vivo, indicate that the lack of ASMase does not affect the pool of satellite cells in healthy muscles, nor their ability to proliferate and differentiate *per se*, nor the normal development of skeletal muscles. However, following damage obtained by the injection of CTX, we found that ASMase-KO mice have a potentially accelerated early regeneration which ameliorates tissue repair process.

Muscle regeneration is a complex event that engages many molecular mediators aimed at regulating the behavior of the different cell types involved in the process, such as inflammatory cells and myogenic precursors cells and whose interaction is essential to restore tissue homeostasis [5]. A functional inflammatory response is mandatory to promote an efficient regenerative process and requires finely regulated infiltration of inflammatory cells and cytokines production [11]. ASMase is critical for cell signaling since, through the ability to generate the second messenger ceramide, it modulates membrane fluidity which is crucial in triggering many cellular processes such as inflammatory pathways [69]. In the injured muscle of ASMase-KO mice we revealed an anti-inflammatory microenvironment marked by: (i) a reduced infiltration of CD45+ inflammatory cells, (ii) an increase of the transcription factor Nrf2 and its downstream genes, and (iii) a decreased gene expression of IL1β and IL6. Accordingly, in muscles, it has been demonstrated that the lack of Nrf2 exacerbates CTX-induced damage and delays the process of muscle regeneration through the induction of a strong inflammatory response [70]. However, the role of Nrf2 in acute muscle damage induced by CTX is still controversial. Using Nrf2-KO mice, it has been recently reported that Nrf2 expression does not affect muscle regeneration [71,72] although some experimental differences, i.e., dosages and types of CTX injected, damaged muscles and days of analysis after injection might explain the discrepancies. In our model, the increase of Nrf2 pathway is transient (3 and 5 days after damage), with higher levels achieved in ASMase-KO mice, thus being concomitant with the dynamic response to the acute inflammation.

When administrated to myoblasts in culture, IL1β inhibits the ability of IGF-1 to promote differentiation into more mature myotubes through the generation of ceramide [73]. In our injury model, the lack of ASMase was accompanied by a reduction of IL1β and an increase of IGF-1 expression, which may be involved in the acceleration of regeneration [58]. These data further confirm that the interplay between the ASMase/ceramide system and cytokine production is tight and multi-faceted. If it is clearly established that inflammatory mediators activate ASMase and this is often mandatory for their intracellular signaling to be effective [33,34,35,36,37], it is also well known that ASMase activation can stimulate cytokine expression and release [38,39,40,41]. As such, we are aware that further investigations are required to clearly dissect the molecular pathway underlying A-SMase inhibition.

In lesioned muscles, the conversion to the anti-inflammatory microenvironment, essential for completing muscle regeneration, is characterized by the switch from M1 (pro-inflammatory) to M2 (anti-inflammatory) macrophages [12]. The molecular/cellular pathways involved in the macrophage phenotype transition during muscle injury/regeneration are still under investigation. Our results show that in ASMase-KO mice the balance between M1 and M2 macrophages is altered toward an M2 phenotype and this is correlated with the increased expression of Nrf2. Moreover, Kobayashi and colleagues have also recently clarified that Nrf2 inhibits the expression of inflammatory cytokines in M1 macrophages, thus blunting the inflammatory response [74]. The analysis of bone marrow-derived macrophages from WT and ASMase-KO mice revealed that the genetic ablation of the protein results in an altered polarization of M1 macrophages towards an M2 phenotype corresponding to a higher expression of Nrf2. Another fundamental pathway in macrophage differentiation and polarization that can somewhat play a role in our system is that of IGF-1. Indeed, the activator of muscle regeneration IGF-1, which we found being increased in ASMase-KO mice after CTX injury, may also reduce inflammation and M2 macrophage polarization [59]. In addition, other pathways are able to direct macrophages polarization towards an M2 phenotype and contribute to muscle healing after damage, as for instance those involving AMP-activated protein kinase and mitogen-activated protein kinase phosphatase-1 [75,76], or the transcription factors C/EBPβ and Nfix [77,78]. The molecular and functional link between ASMase and these signaling events deserves to be investigated since this is the first report on a role for ASMase in muscle regeneration related to macrophage polarization.

Of note, the expression of IL1β and TNF-α was significantly reduced but not completely suppressed in ASMase-KO cells, thus ensuring the beneficial role of the two cytokines in muscle regeneration. Indeed, TNF-α is known to promote myoblast proliferation by activating the transcription factor nuclear factor-kappa B signaling [79] and mice treated with a TNF-α neutralizing antibody and TNF-α receptor double KO mice displayed severe defects in muscle regeneration [80,81]. Regarding IL-1β, it promotes the production of IL6 in skeletal muscle cells, which is fundamental for migration, proliferation and differentiation of myoblast [82].

Finally, we also provide a proof-of-concept that the pharmacological ablation of ASMase activity in M1 macrophages, obtained by the administration of the FIASMA amitriptyline, results in a downregulation of proinflammatory cytokine expression. It is worth noting that the anti-inflammatory effect of FIASMAs has been already demonstrated in different inflammatory diseases [83,84].

In conclusion, our new data demonstrate that blunting ASMase has a beneficial effect in resolving inflammation during muscle damage and thus may account for possible therapeutic usage of FIASMAs in conditions of altered muscle recovery as a consequence of excessive inflammation.

## Figures and Tables

**Table 1:** List of primer sequences used for RT-qPCR analysis.

Name	Primers Sequences
**CD80**	F: 5′-AGTTTCTCTTTTTCAGGTTGTGAA-3′ R: 5′-CACCCGGCAGATGCTAAAGA-3′
**CD163**	F: 5′-CTCCTGTGGACTCTGAAGCG-3′ R: 5′-CTCTGAATGACCCCCGAGGA-3′
**CD206**	F: 5′-ATGGATTGCCCTGAACAGCA-3′ R: 5′-TGTACCGCACCCTCCATCTA-3′
**IL1β**	F: 5′-CCCTGCAGCTGGAGAGTGTGGA-3′ R: 5′-TGTGCTCTGCTTGTGAGGTGCTG-3′
**NOS2**	F: 5′-GTTCTCAGCCCAACAATACAAGA-3′ R: 5′-GTGGACGGGTCGATGTCAC-3′
**F4/80**	F: 5′-TGACTCACCTTGTGGTCCTAA-3′ R: 5′-CTTCCCAGAATCCAGTCTTTCC-3′
**MyoD**	F: 5-CTGGCGCCGCTGCCTTCTAC-3′ R: 5′-GGCCGCTGTAATCCATCATGCCA-3′
**Myogenin**	F: 5′-GACCCTACAGACGCCCACAATC-3′ R: 5′-ACACCCAGCCTGACAGACAATC-3′
**MyHCII**	F: 5′-AAGCGAAGAGTAAG CTGTC-3′ R: 5′-GTGATTGCTTGCAAAGGAAC-3′
**MyHCIV**	F: 5′-ACAAGCTGCGGGTGAAGAGC-3′ R: 5′-CAGGACAGTGACAAAGAACG-3′
**DMD**	F: 5′-GGAAGAAGTAGAGGACTGTTATG-3′ R: 5′-AGGTCTAGGAGGCGTTTTCC-3′
**Myoz1**	F: 5′-GGAACTTGGCATTGACCTACTG-3′ R: 5′-AAACTTGGGCATCTGGAAGG-3′
**TNF-α**	F: 5′-CCCACGTCGTAGCAAACCACC-3′ R: 5′-TCGGGGCAGCCTTGTCCCTT-3′
**Nrf2**	F: 5′-CATTCCCGAATTACAGTGTC-3′ R: 5′-GGAGATCGATGAGTAAAAATGG-3′
**HO-1**	F: 5′-CATGAAGAACTTTCAGAAGGG-3′ R: 5′-TAGATATGGTACAAGGAAGCC-3′
**GPX1**	F: 5′-GGAGAATGGCAAGAATGAAG-3′ R: 5′-TTCGCACTTCTCAAACAATG-3′
**SOD3**	F: 5′-AGAGAGAGTATTTGGGAACC-3′ R: 5′-AAACTAAGCTGCAAAGTCTC-3′
**RPL32**	F: 5′-TTAAGCGAAACTGGCGGAAAC-3′ R: 5′-TTGTTGCTCCCATAACCGATG-3′
**RPL38**	F: 5′-GAAGGATGCCAAGTCTGTCAA-3′ R: 5′-GAGGGCTGGTTCATTTCAGA-3′
**GAPDH**	F: 5′-ACCACAGTCCATGCCATCAC-3′ R:5′- TCCACCACCCTGTTGCTGTA-3′
**36B4**	F: 5′-AGGATATGGGATTCGGTCTCTTC-3′ R: 5′-TCATCCTGCTTAAGTGAACAAACT-3′

## Data Availability

The datasets used to support the findings of this study are included in the present article and are available from the corresponding author upon request.

## Supplementary Materials

The following are available online at https://www.mdpi.com/article/10.3390/cells10113028/s1, Figure S1: Characterization of skeletal muscle phenotype in ASMase-KO mice; Figure S2: Gating strategy to isolate satellite cells from WT and ASMase-KO muscles; Figure S3: Acid sphingomyelinase in muscle regeneration after injury; Figure S4: Acid sphingomyelinase controls muscle regeneration by affecting macrophage polarization.
