# Supplementary material for: Acid Sphingomyelinase Controls Early Phases of Skeletal Muscle Regeneration by Shaping the Macrophage Phenotype
Source: Cells. 2021 Nov 5;10(11):3028. doi: 10.3390/cells10113028 (PMC8616363; doi:10.3390/cells10113028)
Supplement: Supplementary file 1 [file cells-10-03028-s001.zip › cells-1424709-SI.pdf]

Supplementary Materials

# Acid Sphingomyelinase Controls Early Phases of Skeletal Muscle Regeneration by Shaping the Macrophage Phenotype

**Paulina Roux-Biejat [1], Marco Coazzoli [1], Pasquale Marrazzo [2], Silvia Zecchini [1], Ilaria Di Renzo [1], Cecilia Prata [3], Alessandra Napoli [1], Claudia Moscheni [1], Matteo Giovarelli [1], Maria Cristina Barbalace [2], Elisabetta Catalani [4], Maria Teresa Bassi [5], Clara De Palma [6], Davide Cervia [4], Marco Malaguti [2], Silvana Hrelia [2], Emilio Clementi [1,5], Cristiana Perrotta [1,*]**

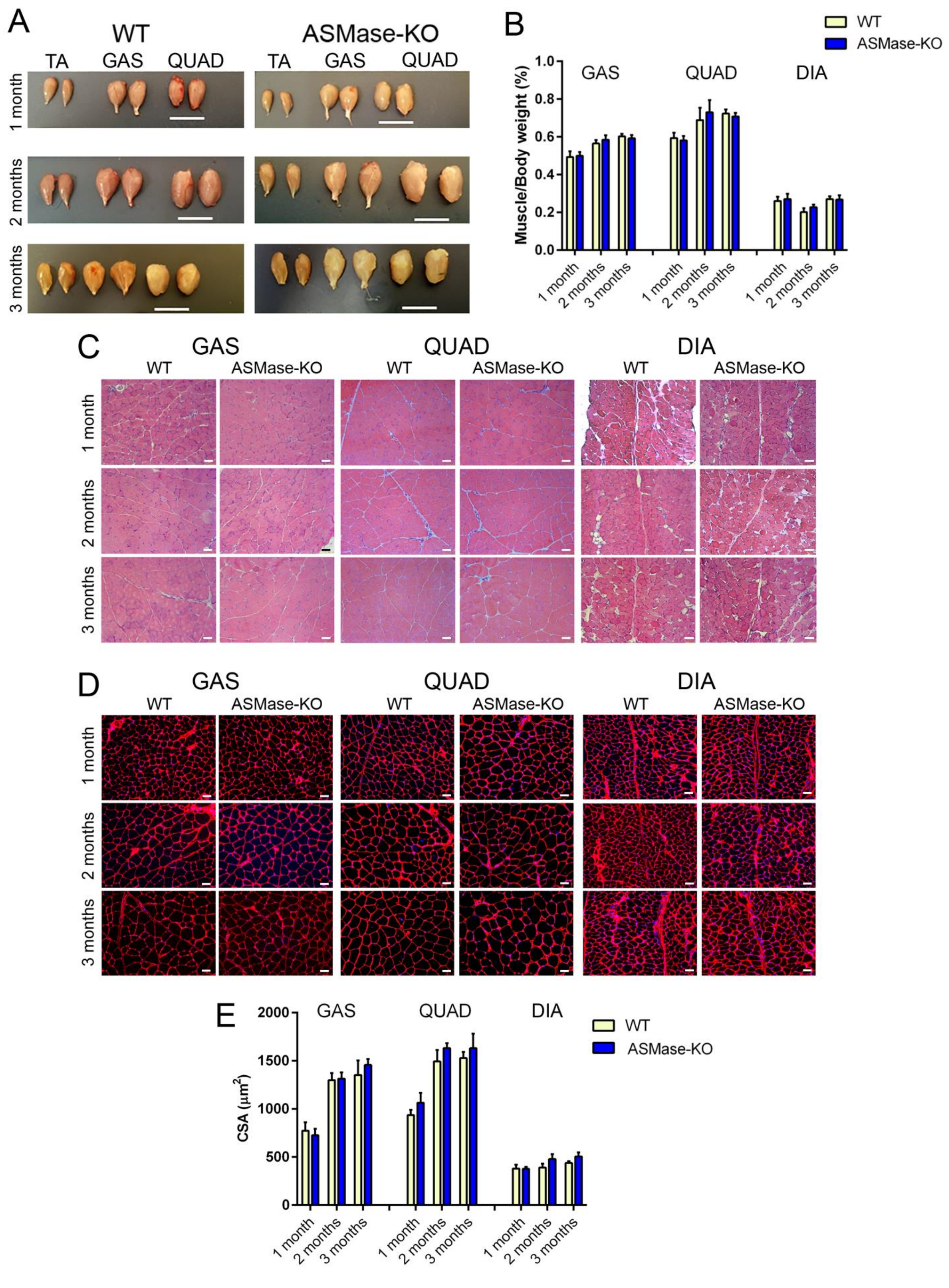


**Supplementary Figure S1.** Characterization of skeletal muscle phenotype in ASMase-KO mice. (**A**) Representative images of Tibias anterior (TA), Gastrocnemius (GAS) and Quadriceps (QUAD) muscles of 1 month, 2 months and 3 months old WT and ASMase-KO

mice. Scale Bar, 10 mm. (**B**) Muscle weight, normalized to body weight, of GAS, QUAD and Diaphragm (DIA) muscles of 1 month, 2 months and 3 months old WT and ASMase-KO mice. (**C,D**) H&E staining (**C**) and Laminin (red) and DAPI (Blue) (**D**) immunostaining of cryosections of muscles of 1 month, 2 months and 3 months old WT and ASMase-KO mice. Scale Bar, 50 μm. (**E**) Quantification of CSA of GAS, QAD and DIA of 1 month, 2 months and 3 months old WT and ASMase-KO mice (n ≥ 4 mice).

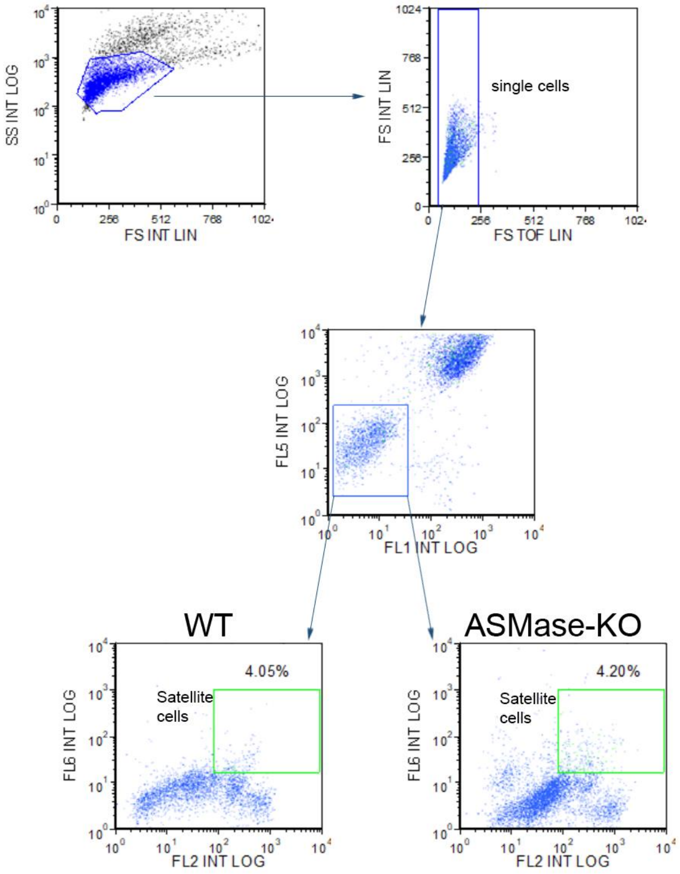


**Supplementary Figure S2.** Gating strategy to isolate satellite cells from WT and ASMase-KO muscles. Satellite cells were identified as an enriched population of $\alpha$7-Integrin-PE (FL-2) and CD34-Alexa Fluor 647 (FL6) double-positive cells and CD45-PE-Cy7, CD31-PE-Cy7 (FL5), CD80-FITC, CD86-FITC, CD14-FITC and Sca1-FITC (FL1) negative cells.

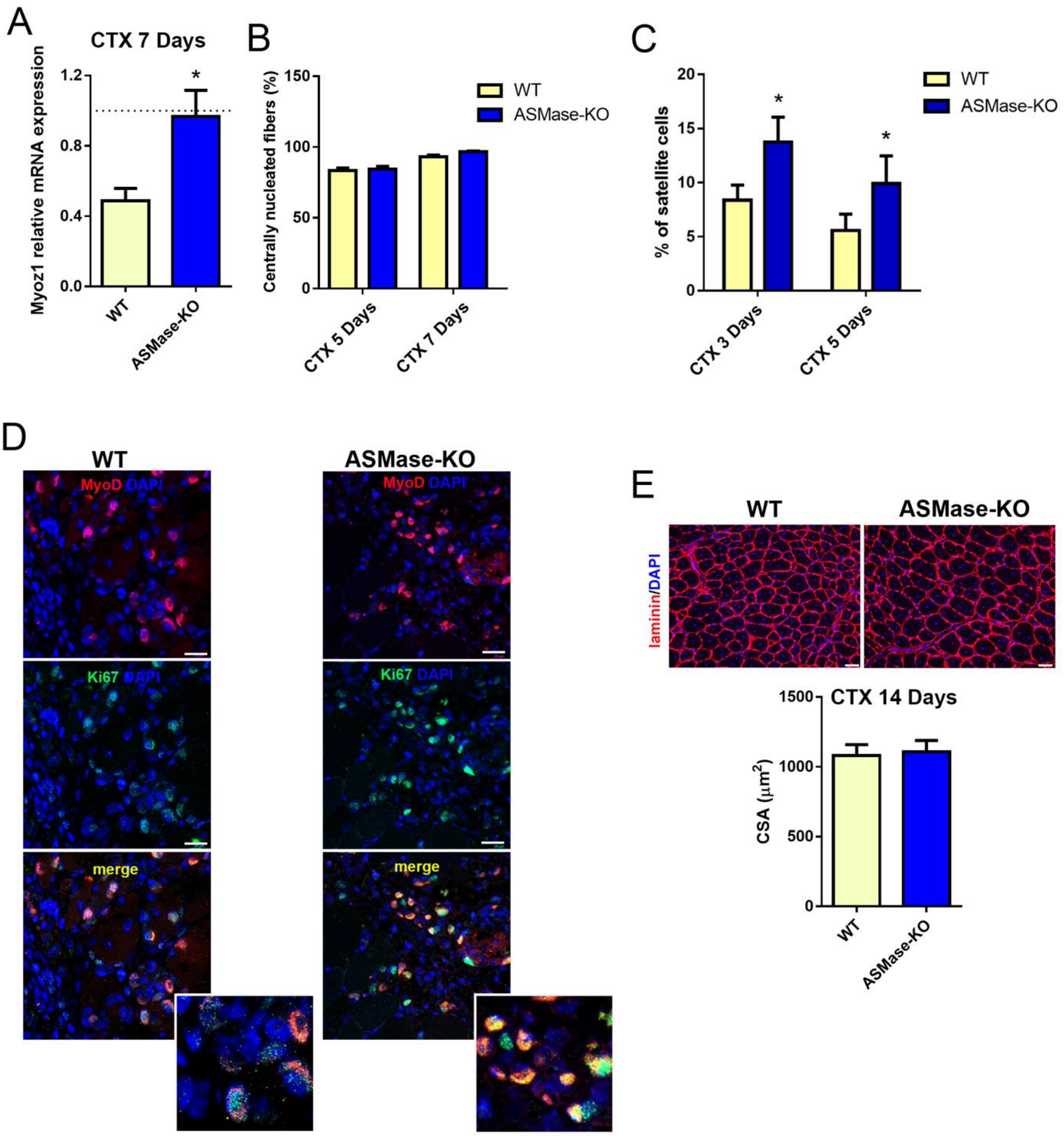


**Supplementary Figure S3.** Acid sphingomyelinase in muscle regeneration after injury. (**A**) RT-qPCR analysis of Myoz1. Values are expressed as mean ± SEM (n = 3 mice) normalized vs the untreated controls (dashed line). * $p < 0.05$, vs the respective WT. (**B**) Quantification of centrally nucleated fibers (%) measured in H&E-stained transversal sections of TA muscles from WT and ASMase-KO mice at 5 and 7 days after CTX injection. Values are expressed as mean ± SEM. (**C**) Satellite cell quantification by flow cytometry in TA muscles of WT and ASMase-KO mice at 3 and 5 days after CTX injection (n ≥ 4 mice). Values are expressed as mean ± SEM. * $p < 0.05$ *vs* the respective WT control. (**D**) Representative images of proliferating satellite cells, *i.e.* MyoD (Red) and Ki67 (green) positive cells, in TA muscles

of WT and ASMase-KO mice at 5 days after CTX injection. Nuclei were counterstained with DAPI (blue). Scale Bar, 20 µm. (**E**). Representative images of laminin immunostaining of transverse sections of TA muscles at 14 days after CTX injection. Nuclei were counterstained with DAPI (blue). Scale bar, 50 µm. The graph shows the mean CSA quantification measured on laminin staining (n = 3 mice). Values are expressed as mean ± SEM.

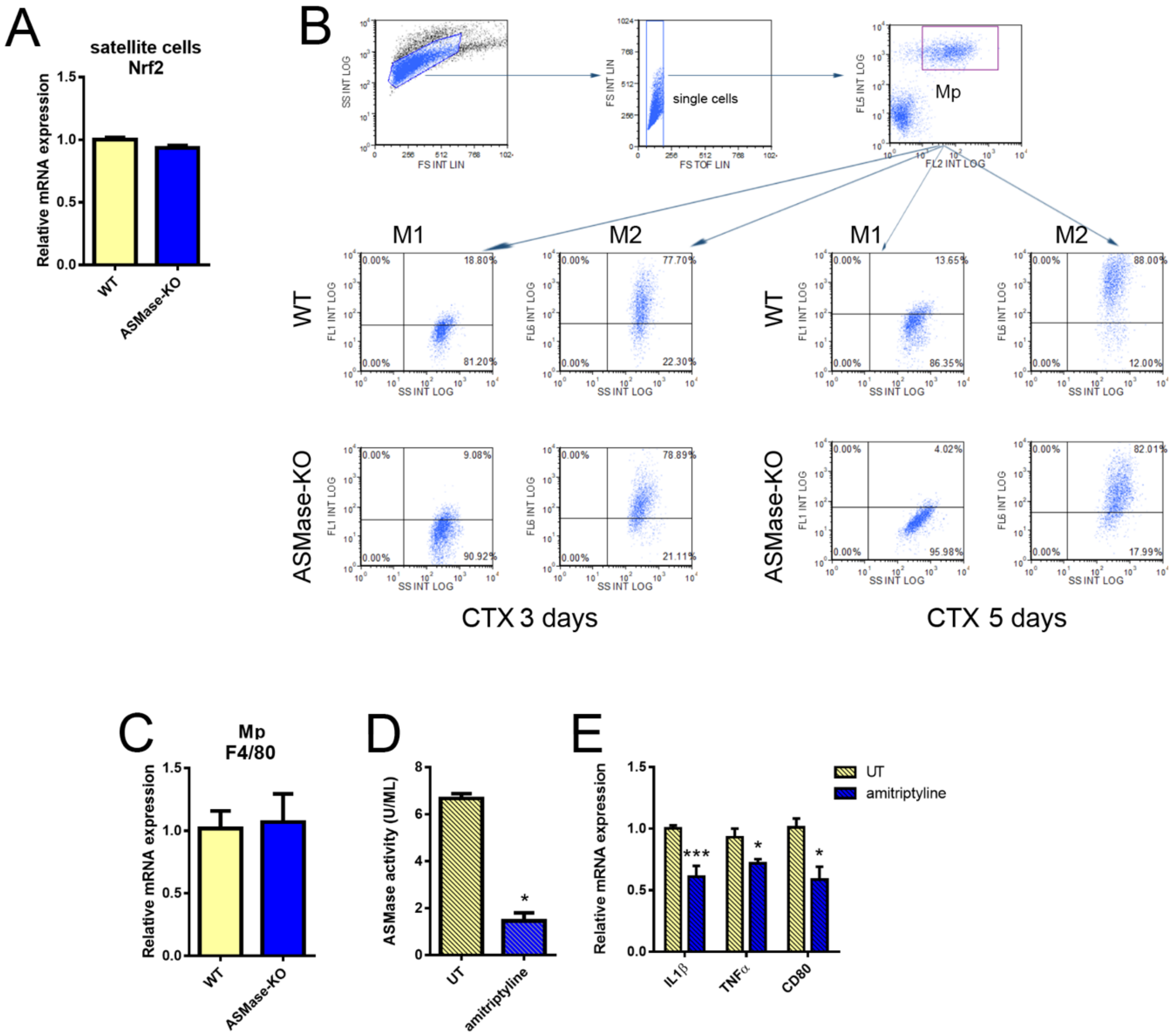


**Supplementary Figure S4.** Acid sphingomyelinase controls muscle regeneration by affecting macrophage polarization. (**A**) RT-qPCR analysis of Nrf2 satellite cells isolated from WT and ASMase-KO mice muscles (n = 4). (**B**) Gating strategy to isolate macrophages from WT and ASMase-KO CTX injured muscles (3 and 5 days). M1 macrophages were identified as CD45-PE-Cy7 (FL5), F4/80-PE (FL2), CD80-FITC (FL1) positive cells; M2 macrophages as CD45-PE-Cy7 (FL5), F4/80-PE (FL2), CD206-APC (FL6) positive cells. (**C**) RT-qPCR analysis of the F4/80 marker in differentiated macrophages (Mp) isolated from WT and ASMase-KO mice muscles (n = 3). Values are expressed as mean ± SEM. (**D**) ASMase activity measured in M1 macrophages from WT mice treated with the FIASMA amitriptyline (5 µM) during

the differentiation. Values are expressed as mean ± SEM (n = 4 mice). * $p < 0.05$ *vs* the untreated control. (**E**) RT-qPCR analysis of the M1 markers IL1β, TNF-α, CD80 in amitriptyline treated M1 macrophages. Values are expressed as mean ± SEM (n ≥ 3 mice). * $p < 0.05$, *** $p < 0.001$ vs the respective untreated control.
